# The Effects of Nitrate on Brown Fat Fraction and Activation in Older Adults With Type 2 Diabetes: A Randomised, Double‐Blind and Placebo‐Controlled Crossover Trial

**DOI:** 10.1002/ejsc.70117

**Published:** 2026-02-20

**Authors:** Rebecca A. Neal, Jo Corbett, Joseph T. Costello, Zoe L. Saynor, Clare M. Eglin, Maria Perissiou, Michael Cummings, Hermione Price, Stephen J. Bailey, S. Sendhil Velan, Suresh Anand Sadananthan, John Totman, Janet Rennell‐Smyth, Anthony I. Shepherd

**Affiliations:** ^1^ Department of Rehabilitation and Sport Sciences Bournemouth University Poole UK; ^2^ Extreme Environments Laboratory School of Psychology Sport & Health Sciences Faculty of Science and Health University of Portsmouth Portsmouth UK; ^3^ School of Health Sciences Faculty of Environmental and Life Sciences Southampton University Southampton UK; ^4^ Clinical Health and Rehabilitation Team, School of Psychology, Sport & Health Sciences Faculty of Science and Health University of Portsmouth Portsmouth UK; ^5^ Diabetes and Endocrinology Department Portsmouth Hospitals University NHS Trust Portsmouth UK; ^6^ Research and Development Tom Rudd Unit Moorgreen Hospital Southern Health NHS Foundation Trust Southampton UK; ^7^ National Centre for Sport and Exercise Medicine, School of Sport, Exercise and Health Sciences Loughborough University Loughborough UK; ^8^ Human Magnetic Resonance Centre Institute of Applied Life Sciences University of Massachusetts Amherst Amherst Massachusetts USA; ^9^ Institute for Human Development and Potential, Agency for Science Technology and Research (A*STAR) Singapore Singapore; ^10^ Fatima College of Health Sciences Abu Dhabi UAE; ^11^ Department of Medical Science & Public Health Faculty of Health & Social Sciences Institute of Medical Imaging & Visualisation (IMIV) Bournemouth University Poole UK; ^12^ Pateint and Public Involvement Member University of Portsmouth Portsmouth UK

**Keywords:** beetroot, brown adipose tissue, infra‐red thermography, MRI, proton density fat fraction

## Abstract

Type 2 diabetes mellitus (T2DM) is a metabolic disease characterised by chronic hyperglycaemia, whereas obesity is a major risk factor which increases morbidity and mortality. Treatments that alter white adipose tissue to express a metabolically active brown adipose phenotype in rats may offer adjunct treatment in people with T2DM. To investigate whether inorganic nitrate supplementation from beetroot juice (BJ) alters brown adipose tissue (BAT) fat fraction and activation in humans. Thirteen older adults with T2DM (glycated haemoglobin [HbA1c]: 58 ± 13 mmol·mol^−1^ and body mass index: 29.1 ± 3.1 kg·m^−2^) completed a double‐blind, randomised, balanced and placebo‐controlled crossover study. Outcome measures (including BAT fat fraction; activation; plasma [nitrate] and [nitrite]) were assessed before and after 14‐day of 140 mL·day^−1^ BJ containing inorganic nitrate (∼12.4 mmol·L^1^) or a placebo (∼0.1 mmol·L^1^). Magnetic resonance imaging (MRI) and infrared thermography (IRT) were performed to image supraclavicular BAT following a rested cooling protocol, consisting of 60‐min exposure via a cold water (8.1 ± 1.2°C) perfused jacket. Respiratory parameters, including respiratory exchange ratio [RER] and mean skin temperature, were measured during the cooling protocol to confirm participants were not shivering. BJ significantly increased venous plasma [nitrate] and [nitrite] versus placebo (*p* < 0.001) but did not affect BAT fat fraction (*p* = 0.650) or activation (*p =* 0.152). Cooling significantly reduced mean skin temperature in BJ (−0.8 ± 0.7°C) and placebo (−0.6 ± 0.6°C) (*p* < 0.001) and RER remained representative of nonshivering thermogenesis throughout (0.88 ± 0.05 a.u.). 14‐day of nitrate supplementation did not increase BAT fat fraction or activation in older adults with T2DM.

## Highlights

- This trial investigated nitrate supplementation on brown adipose tissue fat fraction and activation in adults with type 2 diabetes
- Nitrate supplementation increased plasma [nitrite], whereas a water‐perfused jacket reduced mean skin temperature to investigate brown fat activation via cooling
- Nitrate supplementation did not increase BAT fat fraction or activation
- Possible explanations for the lack of effect include; adiposity, insufficient cooling periods, lack of efficacy of nitrate and hypoglycaemic agents stimulating BAT

**Abbreviations**
BJ: beetroot juice
IRT: infrared thermography

## 1 Introduction

Type 2 diabetes mellitus (T2DM) is a metabolic condition characterised by chronic hyperglycaemia and progressive insulin resistance (Prospective 1995), which is exacerbated by excessive white adipose tissue (WAT) (Wu et al. 2021). With the number of people with T2DM continuing to rise, this pandemic is expected to reach 700 million people by 2045 (Saeedi et al. 2019), such that the costs associated with its clinical management are likely to become unsustainable (Thomas et al. 2016). Therefore, identifying cost‐effective interventions to help weight loss and increase energy expenditure (EE), with the potential to reduce disease risk and progression, is imperative. Interventions that require exercise or lifestyle interventions are efficacious but have demonstrated limited adherence and alternative therapeutic interventions should be explored.

Diets rich in fruits and vegetables are well known to have cardiovascular benefits (Joshipura et al. 1999, 2001; Larsen et al. 2006; Bryan et al. 2007; Govoni et al. 2008; Webb et al. 2008; Kapil et al. 2011; Shepherd et al. 2019) and reduce the risk of developing T2DM (Carter et al. 2010). These cardioprotective diets appear to be particularly effective in humans when rich in green leafy vegetables and beetroot (Bryan N and Hord N 2010; Gilchrist and Benjamin 2010; Dewhurst‐Trigg et al. 2018). This may, at least in part, be due to their high concentration of inorganic nitrate and its beneficial effects on cardiovascular health because of its effect on increasing circulating substrates for nitric oxide (NO•) synthesis (Bryan N and Hord N 2010). Briefly, dietary inorganic nitrate is converted in a reversible stepwise reaction to nitrite via bacteria in the oral cavity (Duncan et al. 1995; L'Heureux et al. 2023). Subsequently, ingested nitrite is reduced to NO• in the stomach (Benjamin et al. 1994). The remaining nitrite, alongside other reactive nitrogen intermediates, such as S‐nitrosothiols, is then absorbed into the circulation, where it acts as a storage pool for subsequent NO• production (Kapil et al. 2020).

WAT is primarily an energy store, whereas brown adipose tissue (BAT) is a metabolically active tissue. People with an elevated BMI are associated with reduced BAT activity (Orava et al. 2011), which makes increasing BAT activity in people with T2DM is a promising therapeutic strategy because it increases glucose disposal (see review here: Maliszewska and Kretowski 2021) and increases EE. BAT is used in human nonshivering thermogenesis (van Marken Lichtenbelt and Schrauwen 2011) for heat production (Cannon and Nedergaard 2004) and is stimulated by cold environments and/or diet in adults and in vivo (van der Lans et al. 2013; Labbé et al. 2015). Indeed, BAT could contribute to ∼46–211 kcal·day^−1^ during mild cold exposure (Carpentier et al. 2018). To produce heat in children (Symonds et al. 2011) during cold exposure in mice (Enerbäck et al. 1997), uncoupling protein (UCP)‐1, an inner mitochondrial protein, is upregulated in BAT, resulting in a reduced mitochondrial proton gradient making the cell less energy efficient. Inorganic nitrate supplementation has also been shown to increase UCP‐1 expression in BAT in rats (Roberts et al. 2015). Increased dietary nitrate intake elevates cyclic guanosine monophosphate [(cGMP)] in vivo (Bryan et al. 2005; Kapil et al. 2011), which is known to increase brown fat expression by ‘beigeing’ WAT in rats (Mitschke et al. 2013), through an NO• dependent process (Roberts et al. 2015). The effect of nitrate beigeing WAT has yet to be investigated in humans. Nitrate rich beetroot juice may offer a thermogenic stimulus to increase daily EE by ‘beigeing’ WAT to BAT in people with increased WAT stores. However, species differences in nitrate metabolism (Montenegro et al. 2016) are profound and the translational potential of these encouraging preclinical data to humans is unclear and in need of empirical investigation. This study sought to introduce ideas from animal evidence and investigate the potential impact of nitrate on brown fat in adults with type 2 diabetes mellitus.

Recent developments in the ability to noninvasively measure BAT activation in healthy humans (Sun et al. 2019) and BAT fat fraction in people with T2DM (Kokura et al. 2007) using magnetic resonance imaging (MRI) has opened the possibility to study the effects of nitrate on BAT activation in humans. Although the more easily accessible technique of infrared thermography (ITR) (Brasil et al. 2020; Vidal et al. 2023) has yet to be used to assess BAT activation in people with T2DM. Utilising in vivo methods to increase BAT mass and thermogenesis may offer treatments for obesity and related metabolic disorders including T2DM. Therefore, the present study aimed to assess the effects of 14‐day inorganic nitrate supplementation on BAT fat fraction and activation in adults with T2DM. We hypothesised that nitrate would reduce BAT fat fraction and increase activation.

## 2 Materials and Methods

### 2.1 Ethical Approval

This double‐blinded, balanced, randomised and crossover control trial was granted a favourable ethics opinion by the NHS, Berkshire B, Research Ethics Committee (21/SC/0378) and the Health Research Authority. This study conforms to the standards of the Declaration of Helsinki, and the study protocol was preregistered on the ClinicalTrials.gov website (ID no. NCT05342012).

### 2.2 Participants

**FIGURE 1:**
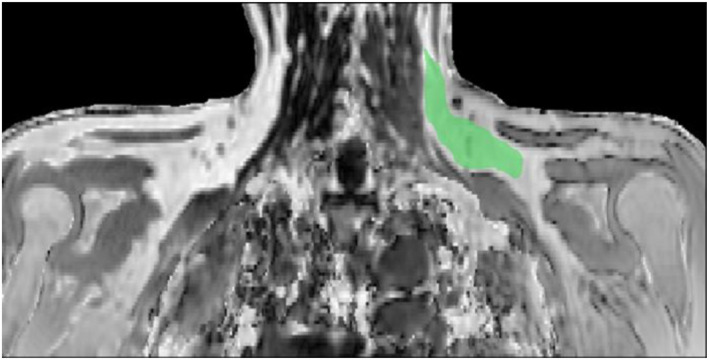
Example magnetic resonance image with left (postcooling) supraclavicular brown fat depot region of interest highlighted green. Images taken pre‐ and post‐60 min seated cooling and 14‐day beetroot intervention.

We recruited people with a clinical diagnosis of T2DM as defined by the World Health Organization. Participants were ≥ 18 years of age and able to provide fully informed written consent. Participants were excluded if they had a body mass index (BMI) > 35, severe claustrophobia, currently smoked (or have stopped within 3 months), used proton pump inhibitors or phosphodiesterase inhibitors (or did not wish to stop using them for the study) or had any other serious medical condition or device that may interfere with data quality and/or participant safety during MRI (e.g., aneurysm clip, cardiac pacemaker/defibrillator, artificial implants, infusion pumps, metallic foreign bodies, surgical screws or similar and permanent piercings). Participants were recruited from primary care (i.e., GP practices), local database of volunteers and via media releases (Table 1.) and their flow through the trial is presented in Figure 1.

**TABLE 1:** Participant characteristics.

	*n* = 13
Age (years)	62 ± 8
Height (m)	1.73 ± 0.08
Mass (kg)	81.4 ± 9.9
BMI (kg·m^−2^)	29.1 ± 3.1
HbA1c (mmol·mol^−1^)	58.2 ± 13.4
Cholesterol (mmol·L^−1^)	4.1 ± 0.8
LDL (mmol·L^−1^)	1.62 ± 0.73
HDL (mmol·L^−1^)	1.5 ± 0.3
Triglycerides (mmol·L^−1^)	2.2 ± 1.1
Non‐HDL (mmol·L^−1^)	2.6 ± 0.7
Chol/HDL (mmol·L^−1^)	2.8 ± 0.5
Metformin	*n* = 10
PPI	*n* = 1
PDE 5i	*n* = 3
GLP‐1	*n* = 3
SGLT2i	*n* = 7
Insulin	*n* = 2

*Note:* Data are presented as means (SD) or *n*. N.B.

Abbreviations: BMI, body mass index; Chol, cholesterol; GLP‐1; glucagon‐like peptide‐1 receptor agonists; HbA1c, glycated haemoglobin; HDL, high‐density lipoprotein; LDL, low‐density lipoprotein; PDE5i, phosphodiesterase type 5 inhibitors; PPI, proton pump inhibitor and SGLT2i, sodium glucose transporter 4 inhibitor.

**FIGURE 2:**
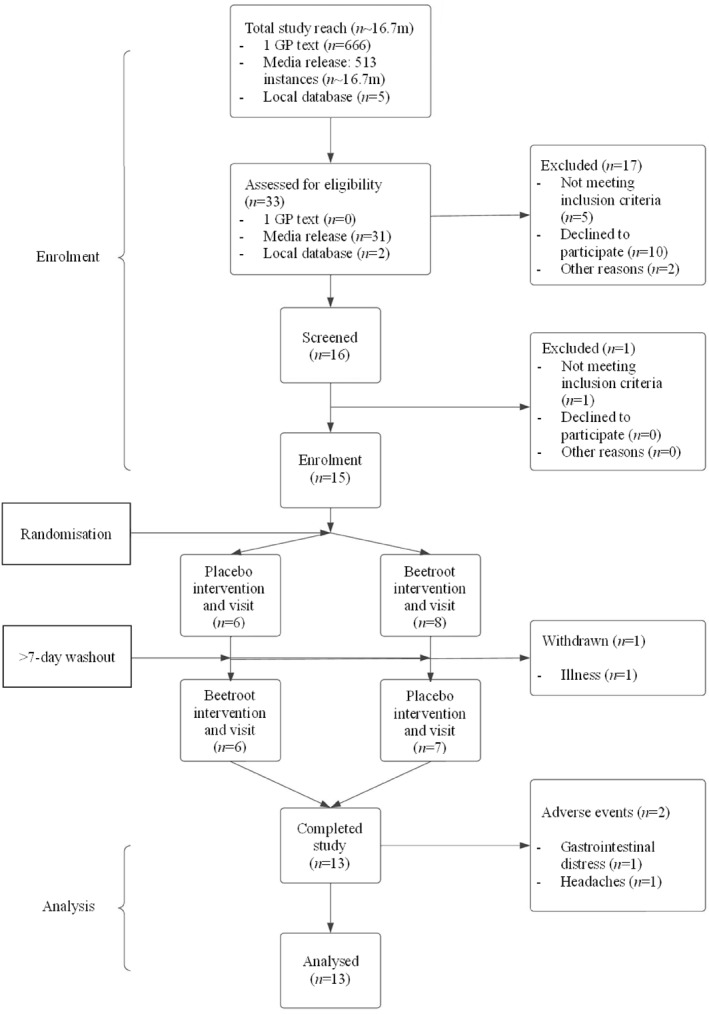
CONSORT diagram. Flowchart of participant progress throughout the cross‐over study.

Participants were randomised, using a computer programme (randomizer.org) by a member of the research team blinded from the supplements, order of testing and had no input into the results, with concealed allocation into one of two experimental conditions to begin in either a control arm of nitrate depleted beetroot juice (placebo) or a nitrate rich beetroot juice (BJ). Participants and outcome assessors were blinded to treatment groups throughout. The placebo appears and tastes identical to BJ and, as previously reported, both have similar antioxidants and polyphenol content (Shepherd, Gilchrist, et al. 2015). Final unblinding of all participants was performed after the creation of a locked analysis data sheet. Data were collected for BAT fat fraction, activation and plasma [nitrate] and [nitrite]. The primary outcome was the change in BAT fat fraction. An a priori sample size calculation was performed to estimate the required *n*. For 80% power, we required 13 people in order to detect a 1 standard deviation change in BAT fat fraction (Holstila et al. 2017). We aimed to recruit 15 people in order to account for 15% dropout rate (Figure 2).

### 2.3 Pre‐Experimental Tests

Participants were provided with a participant information sheet ≥ 48 h prior to providing written informed consent. Participants were fully briefed about the study and provided with the opportunity to ask any questions prior to enrolment. A standard medical history and clinical exam were performed including electrocardiogram (ECG), height, body mass, capillary blood samples from the fingertip and blood pressure. Blood was analysed in house for glycated haemoglobin (HbA1_c_) and lipid profiles (Afinion 2 Analyser, Abbott, Abbott Park, Illinois, United States). Results from the 12‐lead ECG were examined by a clinician alongside anything in the medical history that demonstrated questionable responses and/or contraindications for MRI prior to scanning. Participants were provided with the opportunity to be familiarised with the MRI scanning procedure to assist in screening for claustrophobia prior to commencing the study.

At the end of visit 1 (consent and screening), the correct number of placebo or BJ bottles were placed in opaque bags for participants to take home. Each participant's study number was used to randomly allocate treatment order. Participants were instructed when to take the juice and reminded that mouthwash was prohibited for the duration of the study as it disrupts the oral microbiome responsible for nitrite conversion (Govoni et al. 2008).

### 2.4 Protocol and Outcome Measures

Participants were instructed to ingest 140 mL·day^−1^ of BJ (delivers ∼ 12.4 mmol·L^1^ of inorganic nitrate Beet It, James Whites Drinks Ltd.) or 140 mL·day^−1^ of placebo juice (0.1 mmol·L^1^) in a randomised order for 14‐day prior to visits 2 and 3 and take the last 140 mL dose 1.5 h before arriving at the laboratory. A minimum 7‐day washout period was implemented between visits 2 and 3 in line with our other nitrate trials which have 7 days to be sufficient in humans (Shepherd et al. 2015b, 2015c, 2016; Eglin et al. 2017; Shepherd et al. 2019). The 14‐day intervention was deemed sufficient given that beetroot juice supplementation has previously been shown to improve mitochondrial efficiency (P/O ratio) within 3 days (Larsen et al. 2011) and that exercise training periods also appear to modulate BAT fraction by beiging WAT (Scheel et al. 2022) within 11 days, whereas nitrate supplementation beige WAT after a similar period of time (Roberts et al. 2015).

The primary outcomes were assessed in the Bournemouth Gateway Building, Bournemouth, UK in July 2022 to January 2023. Although the study period was six months, participants completed visits 1 and 2 within 4 weeks, with the minimum 7‐day washout. Participants rested in ambient conditions for 10 min, following which BP was measured and, subsequently, a ∼12 mL venous blood sample drawn from the antecubital fossa into a EDTA K2 (BD) and lithium heparin vacutainers (BD), for determination of plasma [nitrate] and [nitrite]. Participants were familiarised with the environment with 30–60 min in the stable laboratory (22.8 ± 0.8°C and 51.4 ± 9.7% RH) before commencing infrared thermal imaging (described below). Following this, participants wore a cooling jacket for 1 h. A water‐bath (Optima TX150, Grant Instruments Cambridge Ltd, Royston, UK) with cooling coil (C1GR, Grant Instruments Cambridge Ltd, Royston, UK) was prefilled and temperature maintained at 8.1 ± 1.2°C and was used with a water perfused (∼15 m of tubing) jacket (Cool Flow Adjustable Cooling Vest System, Polar Products, Ohio, USA). Water temperature was monitored‐using a thermistor inserted into the bath and‐ambient‐temperature measured using a wet‐bulb‐globe thermometer (WBGT; HT200, Extech Instruments, Knoxville, Tennessee). Pilot testing was conducted to identify the lowest sustainable water temperature for a 60‐min cooling protocol using a commercially available cooling vest. The selected temperature provided a perceptible cold stimulus without inducing shivering thermogenesis. Skin‐thermistors (UU‐VS, Grant Instruments Cambridge Ltd, Royston, UK), connected to a data logger (SQ2040 Squirrel Data Logger, Grant Instruments Ltd, Royston, UK) were attached to the chest (under cooling jacket and vest but not in contact with tubing), arm, thigh and calf to calculate mean skin temperature (Mean *T*_sk_) (Ramanathan 1964; Sun et al. 2019). The thermal image was repeated pre and postcooling on both visits (see details below) and in addition a postcooling MRI scan (3T MRI, Magnetom Lumina, Siemens Heathcare Ltd, Camberley, UK) was commenced within 2–3 min of completion of the cooling protocol.

### 2.5 MRI Imaging of Supraclavicular BAT

MRI of the supraclavicular fat depot was performed to quantify BAT activity. Image slices with 2 mm slice thickness and in‐plane resolution of 1 × 1 mm were acquired using multipoint Dixon sequence (TR = 15 ms, 10 echoes, TE1 = 1.23 ms and delta TE = 1.23 ms) and body matrix coil after anatomical localisation. The images had anatomical coverage of the neck, supraclavicular region and the apices of the lung.

### 2.6 IRT Imaging of Supraclavicular BAT

For acquisition of thermal images (FLIR Systems A320G thermographic camera, UK) taken from dry skin in the supraclavicular and sternal region perpendicular to the camera at 1 m distance (emissivity 0.95), each participant was seated in an upright position (wearing shorts and cotton singlet covering chest skin temperature site but not obstructing IRT measurement) on a chair, with their head in a neutral position looking straight ahead in a room where temperature was kept constant (temperature: 22.8 ± 0.8°C and relative humidity: 51.4 ± 9.8%). Respiratory‐measures of pulmonary oxygen uptake ($\dot{V}$ O_2_), carbon dioxide output ($\dot{V}$ CO_2_) and minute ventilation ($\dot{V}$_E_) were recorded continuously (Cortex Metalyser 3B‐R2, Cortex Medical, Seattle, Washington, USA, calibrated with a two‐point calibration [ambient air; calibration gas, 5% CO_2_, 15% O_2_; with single use turbine flow checked volume measure]) to ensure that any temperature increases were a result of nonshivering thermogenesis as significant changes in these variables is indicative of shivering thermogenesis (Kollias et al. 1972).

### 2.7 Biochemical Analysis

Venous blood samples were placed in a centrifuge and spun at 4000 g, 4°C for 10 min immediately following collection. Once spun, the plasma was pipetted into aliquots with a link anonymised code. The samples were then placed in a −80°C freezer until subsequent analysis. Samples were analysed for [nitrate] and [nitrite] using a Sievers nitric oxide analyser (Sievers NOA 280i, Analytix Ltd, Durham, UK) with our established technique (Shepherd, Gilchrist, et al. 2015; Shepherd, Wilkerson, et al. 2015c, 2016, 2019).

### 2.8 Data Analysis

#### 2.8.1 MRI Analysis

The multiecho complex data obtained using multipoint Dixon sequence were reconstructed using a multiscale graph‐cut algorithm (Berglund and Skorpil 2017). Multiple regions of interest (ROIs) were manually drawn within the left supraclavicular fat depots across approximately 12 image slices per participant on the fat fraction image. Each ROI included approximately 8000 to 10,000 voxels, with a resolution of 1 × 1 × 2 mm. A fat fraction threshold range of 40%–85% was applied to identify BAT based on criteria established in our previous study (Sun et al. 2019). This range was selected to exclude regions with low fat content, such as muscle or bone marrow (< 40%), and regions with high fat content consistent with WAT, which typically exceeds 85% fat fraction in adults (Abe et al. 2021). Thus, this threshold is expected to exclude nearly all WAT within the supraclavicular depot while capturing voxels consistent with BAT. A typical ROI selected within the supraclavicular fat depot is shown in Figure 1, below. The proton density fat fraction (PDFF) of BAT was quantified as the mean fat fraction within the ROIs. Recent developments in the noninvasive measurement of human BAT using MRI (Holstila et al. 2017; Sun et al. 2019) conclude that fat fractions from the BAT regions can be reliably measured using MRI in comparison to PET related measures.

### 2.9 IRT Analysis

Following ∼30 min adjustment to the indoor ambient conditions (22.8 ± 0.8°C and 51.4 ± 9.7% RH) (outdoor conditions July–January 0.4°C–25.5°C) BAT activation was determined by the difference in skin temperature between the SCV fossae region and the sternal control region (Symonds et al. 2012; van der Lans et al. 2014; Vidal et al. 2023). ROI were drawn with FLIR software (FLIR Systems Inc. OR, USA) and saved for each participant following their first visit. This was replicated for each subsequent visit. To ensure this was in the same place, the ROI was fixed from an identifiable landmark, for example, distance from clavicle. IR images were analysed using FLIR ResearchIR software (FLIR Systems Inc. OR, USA). A fixed number of pixels was used for left SCV and control locations with mean and standard deviation temperature taken from these regions, which were assessed to only include relevant tissue within the area. Temperature immediately postcooling was assessed for the following variables: (a) absolute SCV temperatures and (b) SCV—control values.

### 2.10 Statistical Analysis

The distribution of data was assessed using descriptive methods (skewness, outliers and distribution plots) and inferential statistics (Shapiro–Wilk test). Where normal distribution was violated, nonparametric analyses were performed. Results from the Shapiro–Wilk test reported the Δ*T*_sk_ for BJ and placebo VO_2_ and placebo V_E_ were not normally distributed. Paired *t*‐tests were completed to determine differences in dependent variables between beetroot juice and placebo interventions. Two‐way analysis of variance (ANOVA) was completed to determine differences from baseline to end of cooling between interventions. Data are presented as mean (SD) unless otherwise stated. Statistical analysis was performed on GraphPad Prism (version 8) (GraphPad Software, Boston, MA) and statistical difference was accepted as two‐tailed *p <* 0.05.

## 3 Results

In total, 15 participants (Table 1) consented to take part, with 13 completing the study (3 female and 10 male) (Figure 1). Failure to complete the study was due to contraindication for MRI (*n* = 1) and change in health status (*n* = 1). The full anonymised dataset has been made freely available as supplementary material on our University repository.

### 3.1 Protocol Measures

#### 3.1.1 Biomarkers

**FIGURE 3:**
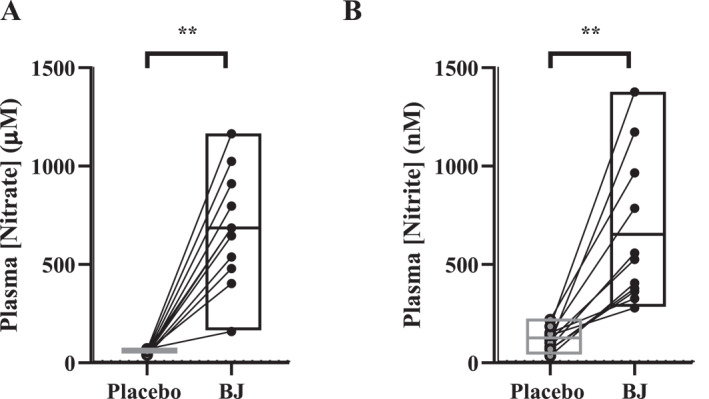
Plasma [nitrate] (A, *n* = 12) and [nitrite] (B, *n* = 11) following a 14‐day placebo and beetroot juice (BJ) intervention. Data are means, minimum and maximum, with individual responses. ** denotes *p* < 0.001.

Plasma [nitrate] and [nitrite] were significantly elevated (*p* < 0.001) following 14‐day BJ compared with placebo (plasma [nitrate] placebo: 53 ± 10 μM and BJ: 682 ± 275 μM and plasma [nitrite] placebo: 120 ± 55 nM and BJ: 649 ± 375 nM; Figure 3).

### 3.2 Cooling

**FIGURE 4:**
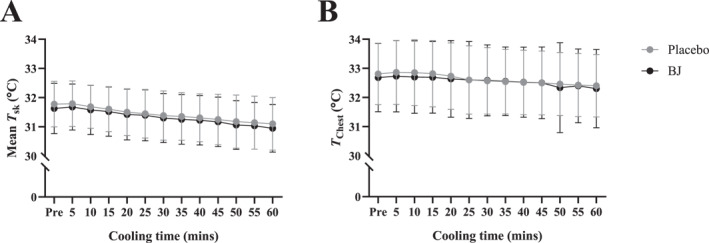
Cooling jacket protocol (mean [SD] *n* = 13). A: Mean skin temperature (*T*_sk_) and B: *T*_sk_ at the Chest, during 60 min seated cooling with a water‐perfused (8.1 ± 1.2°C) jacket following placebo and BJ interventions.

The water‐perfused jacket cooling protocol (Figure 4) significantly reduced mean *T*_sk_ from pre to 60 min in both BJ (−0.8 ± 0.5°C) and placebo (−0.7 ± 0.5°C) interventions (main effect for time, *p* < 0.001) and the change in skin temperature at the chest site located under the cooling jacket was not different between BJ and placebo (*p* = 0.584).

**FIGURE 5:**
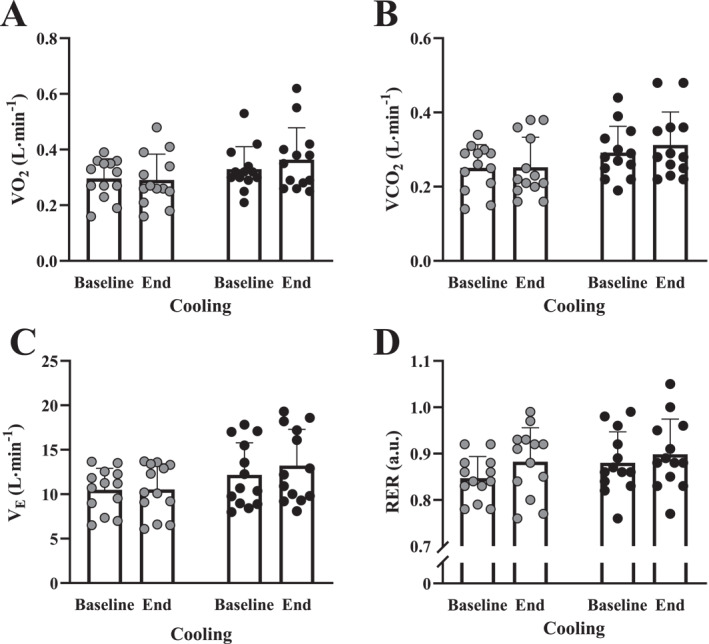
Baseline and end of 60‐min cooling for (A) VO_2_, (B) VCO_2_, (C) V_E_ and (D) RER with individual and mean (SD) data following 14‐day placebo (grey filled circles) and beetroot (BJ: filled circles) interventions (*n* = 13). N.B. BJ: beetroot juice intervention; RER: respiratory exchange ratio; VCO_2_: pulmonary carbon dioxide output; V_E_: minute ventilation and VO_2_: pulmonary oxygen uptake.

There were no main effects for intervention or cooling time or intervention*time interaction effect for $\dot{V}$ O_2_, $\dot{V}$ CO_2_, $\dot{V}$_E_ or RER from baseline to the end of the 60 min cooling and average respiratory variables were not different during cooling following BJ and placebo (*p* > 0.05 and Figure 5).

### 3.3 Outcome Measures

#### 3.3.1 MRI: BAT fraction

**FIGURE 6:**
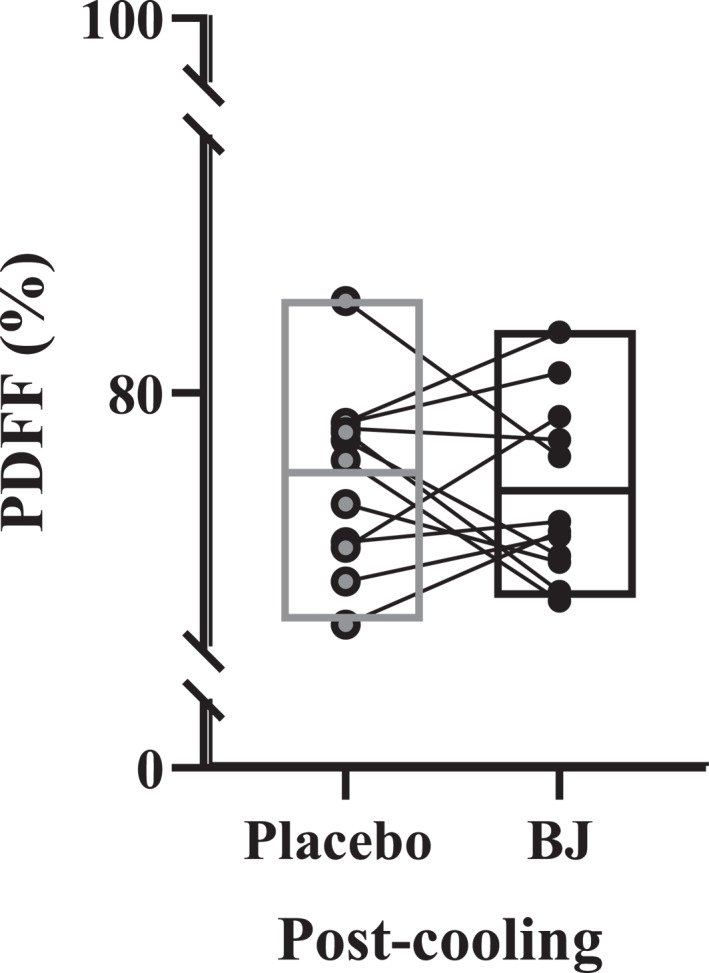
Supraclavicular brown adipose tissue depot proton density fat fraction (PDFF) postcooling following placebo and beetroot Juice (BJ) intervention. Data are means, minimum and maximum with individual data (*n* = 12).

BJ did not change PDFF at the left supraclavicular BAT depot postcooling (73.7 ± 2.7%) compared to the placebo intervention (74.2 ± 2.5%) (*t*_(11)_ = 0.467 and *p* = 0.650; Figure 6).

#### 3.3.2 IRT: BAT activation

**FIGURE 7:**
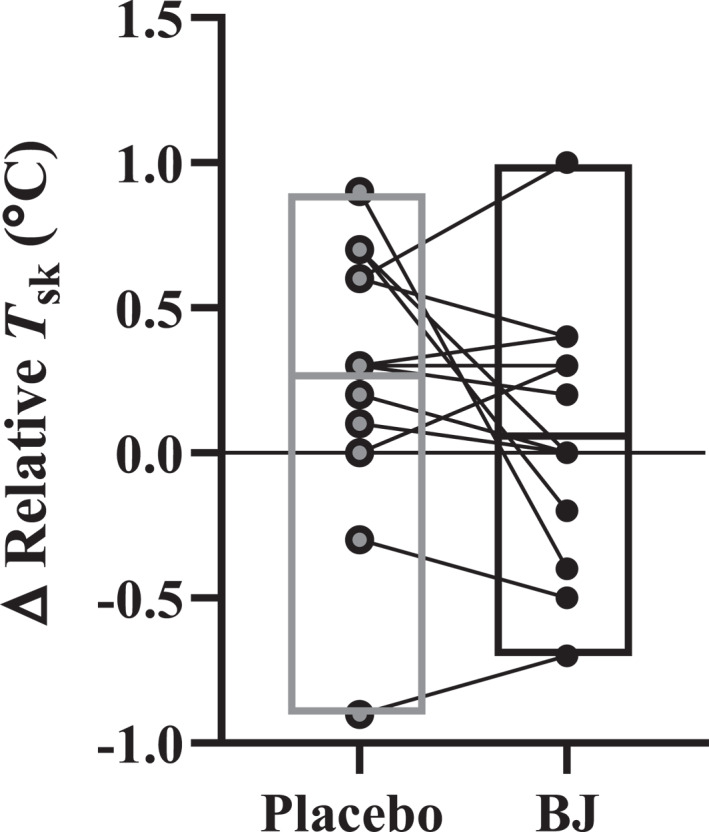
Change in relative skin temperature (*T*_sk_) from infra‐red thermography during a 60‐min cooling protocol following 14‐day placebo (grey filled) or beetroot juice (BJ, filled) intervention (*n* = 13). Data are means, minimum and maximum with individual data.

BJ did not alter the change in relative supraclavicular skin temperature measured via IRT pre to postcooling (0.2 ± 0.5°C) compared to placebo (0.3 ± 0.5°C) (*t*_(12)_ = 1.531 and *P =* 0.152; Figure 7).

## 4 Discussion

This is the first study to assess the effects of dietary nitrate supplementation on brown fat fraction and activation in people with T2DM. The principal novel findings were that, despite a prolonged rise in plasma [nitrite] and using a previously validated cooling protocol, 14‐day of nitrate supplementation (∼12.4 mmol·L·day^1^) had no effect on brown fat fraction or activation (determined via infrared thermography).

### 4.1 Plasma [Nitrate] and [Nitrite]

Plasma [nitrate] and [nitrite] were elevated following 14‐day of nitrate supplementation (∼12.4 mmol·L·day^1^) compared to placebo. This prolonged elevation in circulating plasma [nitrite] increases the potential for NO generation in a nitric oxide synthase (NOS) independent manner. This is particularly important in people with T2DM, given that NO generation is attenuated in conditions of increased oxidative stress (Förstermann and Sessa 2012; Li and Forstermann 2013; Wadley et al. 2013) which is a common characteristic in people with T2DM (Meininger et al. 2000; Xu et al. 2007). The increases in plasma [nitrite] observed in the present study are comparable with our previous work in people with T2DM (Shepherd, Gilchrist, et al. 2015) and also other clinical groups, such as Raynaud's phenomenon (Shepherd et al. 2019) and cold sensitivity (Eglin et al. 2017) with similar dosing protocols. Therefore, it is unlikely that our dosing regimen was the reasons for the lack of effect on beiging WAT. A minimum 7‐day washout period was implemented between visits 2 and 3 in line with our other nitrate trials, which have shown 7 days to be sufficient (Shepherd et al. 2015b, 2015c, 2016; Eglin et al. 2017; Shepherd et al. 2019). However, we cannot preclude the fact that it is possible that there were residual effects of nitrate on BAT fraction, albeit unlikely given that we saw no changes.

### 4.2 BAT Quantity

By increasing the circulating substrate for NO synthesis, we provided the possibility for white fat to beige. Briefly, nitrate supplementation has been shown to increase UCP‐1 expression in BAT (Roberts et al. 2015), which is likely due to an increase in [cGMP] (Bryan et al. 2005; Kapil et al. 2011); increases in [cGMP] have been shown to beige white fat in mice (Mitschke et al. 2013) through an NO• dependent process (Roberts et al. 2015). However, until the present study, this effect had yet to be explored in humans. For the first time, we show that 14‐day of nitrate supplementation (∼12.4 mmol·L·day^1^) sufficient to elevate plasma [nitrate] and [nitrite] does not increase BAT fat fraction (i.e., < PDFF) or activity in humans (specifically in people with T2DM). This indicates that ∼12.4 mmol·L·day^1^ of nitrate supplementation could not beige white fat to express a brown fat phenotype and is unlikely to be a viable adjunct treatment for people with T2DM. However, plasma [nitrate] and [nitrite] are increased after nitrate supplementation in all studies, this is not always the case for plasma [cGMP] (Østergaard et al. 2023). It is possible that we did not increase [cGMP].

Position emission tomography (PET) scanning is the primary tool used to image BAT in humans. More recently, BAT imaging via MRI has been shown to be valid and reliable (Holstila et al. 2017; Yu et al. 2021). Indeed, MRI may be preferred to PET (Franssens et al. 2016, 2017) due to (1) its nonionising radiation approach, which permits multiple measurements, (2) it does not require metabolic activity (Hamilton et al. 2011), (3) it is widely available and (4) it has excellent resolution (Hamilton et al. 2011; Holstila et al. 2017). Specifically, the PDFF analysis has been used favourably in healthy adults (Chen et al. 2013; Lundström et al. 2015; Franssens et al. 2016; Gifford et al. 2016; Romu et al. 2016; Franssens et al. 2017; Koskensalo et al. 2017; McCallister et al. 2017; Franz et al. 2018) based on its robustness and standardisation (Franssens et al. 2016, 2017; McCallister et al. 2017; Franz et al. 2018). Previous research has only measured BAT volume and radiodensity following cold exposure in people with T2DM with PET but not MRI (Blondin et al. 2015). Limited research has used MRI (van Eyk et al. 2020) or MR spectroscopy (Franssens et al. 2017; Koksharova et al. 2017) to determine BAT quantity in people with T2DM, without cold activation, and we also show that the BAT regions in the supraclavicular fat depot could clearly be detected, and BAT quantified through a fat fraction MRI technique.

### 4.3 BAT Activation

We show that 14‐day of nitrate supplementation (∼12.4 mmol·L·day^1^) does not increase BAT activation. Briefly, possible explanations for the lack of effect include: (1) an insufficient cooling protocol; (2) the IRT measurements of relative skin temperature may be insufficient to elucidate potential changes in brown fat activation or (3) that nitrate does not increase BAT in people with T2DM.

In order to stimulate BAT activation, we utilised a skin cooling stimulus. Our intervention was effective at cooling the mean skin temperature over 60 min, but no additional activation of brown fat was observed via IRT. The statistically significant reduction in mean skin temperature may not be a sufficient stimulus for elicit BAT activation and determine any meaningful effects using IRT. Briefly, the IRT imaging technique involved the comparison of sternal (control region) and the supraclavicular region (BAT deposit). Sun et al. [34] activated BAT with a cooling vest at a higher temperature (14.5°C) than the present study (8.1°C) to elicit an increased skin temperature at the SVF measured via IRT from an extended exposure (2 h). Previously, a relatively small sample in adults (*n* = 7) suggested that cooling could activate BAT and be measured through IRT, demonstrated with a 0.2°C increase in supraclavicular skin temperature, above the BAT depot, compared to sternal region (Symonds et al. 2012). However, recent research in a large sample of young participants, with a much lower BMI (*n* = 94, age: 20.5 ± 4.1 years and BMI: 21.5 ± 3.2 kg·m^−2^) from our group corroborates the current findings and no differences between relative or absolute changes in skin temperature at these locations following a cooling (15 min hand 15°C water immersion) protocol (Vidal et al. 2023).

Potential explanations for the lack of effect seen in BAT activation are multifactorial. It is possible that this noninvasive assessment of heat production via BAT with IRT may be insufficient to detect changes in brown fat activation/thermogenesis particularly in older individuals with T2DM (especially with a high BMI). However, our cooling protocol and population (60‐min cooling jacket, 8°C) were different to previous published protocols and thus a greater stimulus, potentially with the addition of a fan, may yield different results. However, cooling people to a greater extent is unlikely to be a viable activity that would be tolerated. One possible explanation for the lack of activation in BAT could simply be that we were looking for changes in the supraclavicular area. Recent evidence using pharmacological stimulation of BAT in older adults (≥ 60 years) show increased 18FDG glucose uptake in the perineal area but not the supraclavicular area (Gacula et al. 2025). Increasing BAT activity in people with T2DM is a promising therapeutic strategy because it increases glucose disposal (Maliszewska and Kretowski 2021). However, cold exposure and elevated [insulin] (Orava et al. 2013) have lower impact on BAT activity (Ouellet et al. 2011; Chondronikola et al. 2016) in people with obesity compared to controls. However, our cohort are overweight (BMI: 29.1 ± 3.1); nonetheless, their body composition maybe at least in part to explain for the lack of effect seen on BAT activation. Finally, it is possible that certain hypoglycaemic agents (van Eyk et al. 2020) already activate BAT and therefore other interventions may struggle to increase this further.

### 4.4 Strengths, Limitations and Future Directions

A key strength of the present study was the study design (randomised, double‐blind, crossover and controlled trial), which is the first to examine the effects of 14‐day of nitrate supplementation on BAT fat fraction and activation in humans (specifically, people with T2DM). However, several limitations warrant discussion. In the present study, diet was not controlled, instead opting to examine the free‐living effects of the intervention (i.e., strong ecological validity), however with this some control is recognised to be sacrificed. Given the large change in dietary [nitrate] and [nitrite] between the active and placebo conditions, this is likely to have had minimal effects on the outcome measures of interest. However, we only assessed BAT activation in an isolated region; therefore, we cannot exclude activation elsewhere in the body. Although MRI provided a more accessible measure of BAT as opposed to PET, the use of PDFF in this instance is limited by a lack of validation of this measure in people with T2DM, so caution should be taken when interpreting these data. Following quality assurance testing of our MR scanner, we identified poor signal quality with one of our coils. We chose to present data only for the left side of the body to ensure excellent quality data.

## 5 Conclusion

This study was the first to assess the effects of inorganic nitrate supplementation on BAT fat fraction and activation in humans. This study successfully imaged brown fat using MRI (determined using PDFF) in people with T2DM. Overall, despite a rise in plasma [nitrite] and an effective skin cooling protocol, BAT activation did not change compared to placebo. Possible explanations for the lack of effect include adiposity, insufficient cooling periods, lack of efficacy of nitrate, hypoglycaemic agents stimulating BAT and a lack of dietary control.

## Author Contributions

R.N., J.C., J.C., C.E., S.V., J.T., J.R.S., A.S. and conceived and designed research: R.N., M.C., H.P., A.S. recruited participants: R.N., A.S., S.B. performed experiments: R.N., S.V., S.S. analysed data. R.N., A.S. interpreted results of experiments: R.N. prepared figures: R.N., S.V., S.S., J.T., A.S. drafted manuscript” R.N., J.C., J.C., Z.S., C.E., M.P., M.C., H.P., S.B., S.V., S.S., J.T., J.R.S., A.S. edited and revised manuscript: R.N., J.C., J.C., Z.S., C.E., M.P., M.C., H.P., S.B., S.V., S.S., J.T., J.R.S., A.S. approved final version of manuscript.

## Funding

This work was supported by the Institute of Medical Imaging and Visualisation at Bournemouth University.

## Conflicts of Interest

The authors declare no conflicts of interest.

## Data Availability

The data that support the findings of this study are openly available in BORDaR at https://doi.org/10.18746/bmth.data.00000388, reference number 388.
